# Up-regulation of miR-224 promotes cancer cell proliferation and invasion and predicts relapse of colorectal cancer

**DOI:** 10.1186/1475-2867-13-104

**Published:** 2013-10-23

**Authors:** Guang-jun Zhang, He Zhou, Hua-xu Xiao, Yu Li, Tong Zhou

**Affiliations:** 1 The First Department of General Surgery, The Affiliated Hospital of North Sichuan Medical College, Nanchong, Sichuan, People’s Republic of China; 2 Institute of Hepatobiliary, Pancreas and Intestinal Disease, North Sichuan Medical College, Nanchong, Sichuan, People’s Republic of China; 3 Department of Pathology, The North Sichuan Medical College, Nanchong, Sichuan, People’s Republic of China; 4 Department of Microbiology and Parasitology, North Sichuan Medical College, Nanchong, Sichuan, People’s Republic of China

**Keywords:** Colorectal cancer, miR-224, SMAD4, Invasion, Relapse

## Abstract

**Background:**

MicroRNAs (miRNAs) are small, non-coding RNAs that can function as oncogenes or tumor suppressors in human cancer. Abnormally expressed miR-224 was found to play a fundamental role in several types of cancer. The aim of this study was to investigate the prognostic and biological values of miR-224 in colorectal cancer (CRC).

**Methods:**

Quantitative RT-PCR (qRT-PCR) was used to evaluate expression levels of miR-224. The postoperative survival rate was analyzed with Kaplan–Meier method. The roles of miR-224 in cell proliferation, migration and invasion were analyzed with pre-miR-224 transfected cells. In addition, the regulation of SMAD4 by miR-224 was evaluated by qRT-PCR, Western blotting and luciferase reporter assays.

**Results:**

In the present study, we demonstrated that miR-224 was significantly up-regulated in CRC tissue samples and associated with disease relapse and a relative poorer disease-free survival rate. Moreover, ectopic expression of miR-224 potently promoted tumor cell proliferation, migration and invasion in vitro. Furthermore, the over-expression of miR-224 in CRC cell lines decreased SMAD4 expression at the translational level and decreased SMAD4-driven luciferase-reporter activity.

**Conclusions:**

Our data suggest that miR-224 could play an oncogenic role in the cellular processes of CRC and represent a novel biomarker for tumor relapse of CRC patients.

## Introduction

Colorectal carcinoma (CRC) is one of the most common cancers, and is a significant contributor to cancer death [1]. Although surgery currently offers the possibility of prolonged survival for CRC patients, a significant number of patients with CRC who undergo curative surgery develop local recurrence or distant metastasis, leading to shorter survival [2]. A better understanding of the molecular mechanisms underlying tumor recurrence or metastasis is essential to facilitate the prevention and treatment of advanced CRC.

MicroRNAs (miRNAs) are endogenous non-coding RNAs that negatively regulate target gene expressions by binding to 3′-untranslated region (UTR) [3,4]. MiRNAs participate in gene regulation, apoptosis, hematopoietic development, the maintenance of cell differentiation, and tumor genesis [5,6]. The dysregulation of miRNAs is common in various carcinomas and plays an important role in tumorigenesis, tumor progression, metastasis and relapse in cancers [7-9].

Recently, miR-224 has been shown to be up-regulated in cervical cancer and pancreatic ductal adenocarcinomas [10,11], and the involvement of miR-224 in the tumorigenesis and development of breast cancer and hepatocellular carcinoma has also been reported [12,13]. Previous reports revealed that miR-224 was upregulated in CRC by miRNA microarray analysis [14,15]. Moreover, miR-224 is one of the most highly differentially expressed miRNAs in methotrexate-resistant cells, and its over-expression induces the resistant phenotype in HT29 colon cancer cells [16]. Taken together, these studies suggest that miR-224 functions as an oncogenic miRNA. However, the association between miR-224 and relapse of colorectal cancer has not been evaluated yet, and the biological roles of miR-224 in CRC remain poorly understood.

Thus, we investigated the relationship between expression level of miR-224 and prognosis in CRC, and further studied the possible function of miR-224 in the CRC cell line. Our study results showed the high expression level of miR-244 in CRC was significantly associated with a relative poorer disease-free survival rate. Moreover, we also demonstrated miR-224 promoted proliferation, migration and invasion of SW480 cells, at least partially through suppression of SMAD4 expression.

## Materials and methods

### Patients and tissue samples

A total of 108 stage I-II (UICC, 6th ed., 2002) colorectal patients received radical surgery at the First Department of General Surgery, the Affiliated Hospital of North Sichuan Medical College, from January 2004 to January 2009, were collected. All clinicopathological characteristics of patients with disease relapse (n=40) or without disease relapse (n=68) within 3 years after surgery were available for all patients. Disease relapse was defined as local recurrence or distant metastasis of colorectal cancer. All tissue specimens were derived from 108 patients who did not received neoadjuvant therapy before surgery. The patients who received postoperative adjuvant therapy were also excluded. To test whether miR-224 was differentially expressed between paired tumor and adjacent normal tissue in the same subject, we recruited a second cohort comprising 20 CRC patients. All tissue samples were immediately frozen in liquid nitrogen and stored at -80°C for subsequent analysis. The median follow-up time was 48.3 months (12.6–62.5 months) until June, 2012. Disease-free survival (DFS) was calculated from radical surgery to the first disease relapse. Informed written consent was obtained from each patient, and research protocols were approved by the Medical Ethics Committee of North Sichuan Medical College.

### Cell culture

The human CRC cell line SW480 was purchase from American Type Culture Collection. The cells were maintained in Dulbecco’s modified Eagle’s medium supplemented with 10% fetal bovine serum, 100 u/ml penicillin and 100 mg/ml streptomycin, at 37°C in a humidified atmosphere of 5% CO_2_.

### RNA extraction and real-time RT–PCR

Total RNA was extracted using TRIzol reagent (Invitrogen, Carlsbad, CA, USA). The PCR primers for miR-224 and U6 were purchased from Applied Biosystems (Applied Biosystems, Foster City, CA, USA). The PCR primers for SMAD4 were 5′-TGGCCCAGGATC AGTAGGT-3′ and 5′-CATCAACACCAATTCCAGCA-3′.The primers for *β*-actin: 5′-CCAAGGCCAACCGCGAGAAGATGAC-3′ and 5′-AGGGTACAT GGTGGTGCCGCCAGAC-3′. The first-strand cDNA was synthesized using the PrimeScript RT reagent Kit (TaKaRa, Dalian, China). Real-time PCR was performed using SYBR Premix Ex Taq (TaKaRa) and measured in a LightCycler 480 system (Roche, Basel, Switzerland). U6 or *β*-actin was used as internal control. Relative gene expression was calculated using 2^-ΔCT^ method, and fold change of gene was calculated using the equation 2^-ΔΔCT^.

### Transfection of miRNA

Ectopic expression of miR-244 in cells was achieved by transfection with Pre-miR-224 precursor (pre-miR-224) (Ambion, Foster City, CA, USA) using Lipofectamine 2000 (Invitrogen). 2 ×10^5^ cells were seeded into each well of a 6-well plate and transfected for 24 h or 48 h. Transfected cells were used in further assays or RNA/protein extraction.

### MTT assay

2×10^4^ SW480 cells were plated onto 96-well plates for 24 h. The cells were then transfected with 50 nM pre-miR-224 or pre-miR-nc. At different time points (24 h, 48 h and 72 h), the culture medium was removed and replaced with culture medium containing 10μl of sterile MTT dye (5 mg/ml). After incubation at 37°C for 4 h, the MTT solution was removed, and 150μl dimethyl sulfoxide (DMSO) was added to each well followed by measuring the absorbance at 570 nm on an enzyme immunoassay analyzer (Bio-Rad).

### Migration and invasion assays

For migration assays, 5×10^4^ cells transfected with either pre-miR-224 or pre-miR-nc were placed into Boyden chambers (Corning, Cambridge, MA, USA) with an 8.0mm pore membrane. For invasion assays, 5×10^4^ cells were placed into chambers coated with 150μg of Matrigel (BD Biosciences, Bedford, MD, USA). Medium containing 10% fetal bovine serum in the lower chamber served as the chemoattractant. After the cells were incubated for 48 h at 37°C in a humidified incubator with 5% CO_2_, the cells remaining on the upper surface of the membranes were removed, whereas the cells adhering to the lower surface were fixed, stained with hematoxylin and counted under a microscope at a magnification of 400× to calculate their relative numbers.

### Western blot analysis

Immunoblotting was performed to detect the expression of SMAD4 in CRC cell lines. Transfected cells were lysed in RIPA lysis buffer (ProMab Biotechnology). Protein was loaded onto a SDS-PAGE minigel and transferred onto PVDF membrane. After probed with 1:500 diluted mouse polyclonal SMAD4 antibody (Santa Cruz Biotechnology, Santa Cruz, CA, USA) at 4°C overnight, the blots were subsequently incubated with HRP-conjugated secondary antibody (1:5000). Signals were visualized using ECL Substrates (Millipore, MA, USA). GAPDH was used as an endogenous protein for normalization.

### Luciferase assay

For luciferase reporter experiments, the wild-type and mutated 3′UTR of SMAD4 mRNA were subcloned into the *Xho*I and *Not*I site of the psicheck-2 vector (Promega) and the new vectors were named psicheck-2-SMAD4-WT and psicheck-2-SMAD4-MUT, respectively. The primers as shown in Table 1 were used to amplify specific fragments. For reporter assay, HEK 293T cells were plated onto 24-well plates at 2×10^4^ cells/well and transfected with 200 ng of psicheck-2-SMAD4-WT or psicheck-2-SMAD4-MUT and 40 nM pre-miR-224 or pre-miR-nc using Lipofectamine 2000 (Invitrogen). Firefly luciferase was used to normalize the Renilla luciferase. After transfection for 48h, cells were harvested and assayed with Dual-Luciferase Reporter Assay System (Promega) according to the manufacturer’s protocols.

**Table 1:** PCR primers and oligonucleotide sequences of constructs in luciferase reporter assay

**Targets**	**Type**	**Primers**	**Oligonucleotide sequence (5′-3′)**
SMAD4			
(site 1, site 2)	WT1	Sense	CACAACTCGAGAGGCACAAGGTTGGTTGCTA
		Antisense	GGAAAAAAGCGGCCGCGACCTTCTGAGCAAGGCAGT
SMAD4	WT2	Sense	CACAACTCGAGTGTGTGACACCACCCTCCTA
(site3)		Antisense	AAGGAAAAAAGCGGCCGCTCAATCCAAGCCCGTGAGTC
SMAD4	MUT1	Sense	TGATGCACTGAATTTTTGGTATAATGTTTAAATCATGT
(site1)		Antisense	CCAAAAATTCAGTGCATCAAATCAAGTACAAAAATA
SMAD4	MUT2	Sense	TGGCACACTGAATGTATAGAGAATTTAAGTAGAAAAGTT
(site2)		Antisense	TATACATTCAGTGTGCCAATTGATATGATCATTGATGG
SMAD	MUT3	Sense	GATTAACACTGAATGGCTGGATCATTCAGAGCTCTCTTCT
(site3)		Antisense	GCCATTCAGTGTTAATCAAAATGGACCTAAAAAGAGCCA

### Statistical analysis

All data presented in this study have been repeated at least three times from three independent experiments. Continuous variables were expressed as the mean ± standard deviation. Measurement data were analyzed using Student's *t*-test, while categorical data were studied using chi-square test. Receiver operating characteristic (ROC) curve was used to determine the cut off value of miR-224 expression. The postoperative survival rate was analyzed with Kaplan–Meier method, and differences in survival rates were assessed with log-rank test. All statistical analyses were performed using SPSS 16.0 software (SPSS, Chicago, IL, USA). Two-sided *P*-values were calculated, and differences were considered significant at *P*-values of <0.05.

## Results

### Patients’ characteristics

A total of 108 patients were included in this study with 40 patients in relapse group (relapse within 3 years after radical surgery) and 68 patients in non-relapse group (no relapse within 3 years after radical surgery). There were no differences between the two groups in terms of age, gender, tumor location, differentiation and TNM stage. The details were seen in Table 2.

**Table 2:** Clinicopathological characteristics of colorectal cancer patients

	**Relapse (n=40)**	**Nonrelapse (n=68)**	***p*-Value**
Age (years)	66(35–82)	66(31–81)	0.576
Gender			
Male	24	47	0.335
Female	16	21	
Location			0.245
Colon	12	28	
Rectum	28	40	
Differentiation			0.318
Well, moderate	22	44	
Poor, mucinous	18	24	
TNM stage			0.119
I	5	17	
II	35	51	

Well: well-differentiated adenocarcinoma,moderate: moderately differentiated adenocarcinoma, poor: poorly differentiated adenocarcinoma, mucinous: mucinous carcinoma.

### Correlations between miR-224 expressions and disease relapse

**Figure 1:**
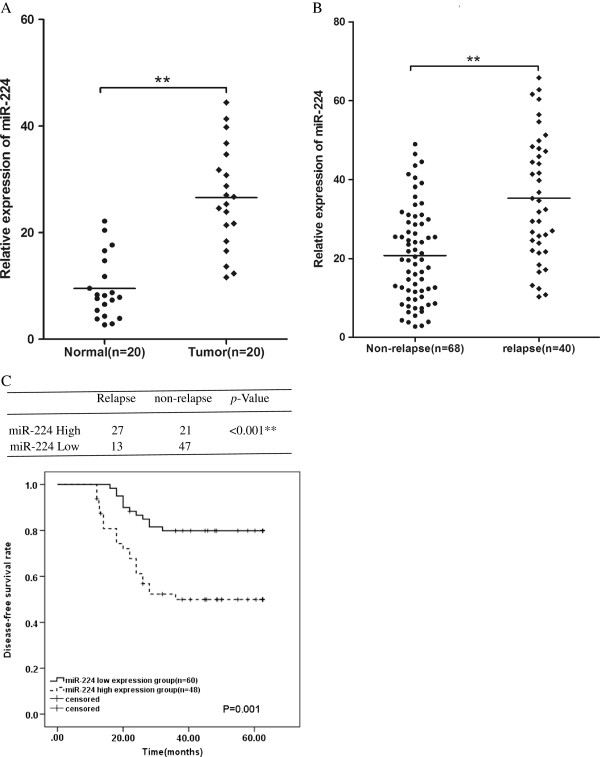
**miR-224 is up-regulated in CRC tissues and associated with the relapse of CRC. (A)** The expression of miR-224 in CRC tumor tissues was significantly higher than in their paired normal colorectal tissues. **(B)** The average expression level of miR-224 was higher in relapse group (n=40) than that in non-relapse group (n=68). **(C)** Chi-square test and Kaplan-Meier analysis were used to demonstrate that high expression of miR-224 was significantly associated with disease relapse (*P*<0.001) and a relative poorer disease-free survival rate (*P*=0.001). **P<0.01.

In this study, we found that miR-224 expression in tumor tissues was significantly higher than that in normal tissues (*P*<0.01, Figure 1A). Using the samples from the second cohort, we found that the miR-224 expression levels were significantly up-regulated in the tissues of CRC patients with disease relapse (n=40) compared with those without disease relapse (n=68) (*P*<0.01, Figure 1B).The expression levels of the miR-224 were categorized as low or high in relation to the cutoff value (25.72) on the basis of ROC curve analysis. Therefore, 48 patients were included in the high expression group and 60 in the low-expression group. Among patients with miR-224 high expression, 27 patients relapsed (27/48, 56.3%), while only 13 patients relapsed (13/60, 21.7%) among patients with miR-224 low expression. Using chi-square test and Kaplan-Meier analysis, the results demonstrated that high miR-224 expression was significantly associated with disease relapse and a relative poorer disease-free survival rate (Figure 1B).

### MiR-224 promotes CRC cell proliferation

**Figure 2:**
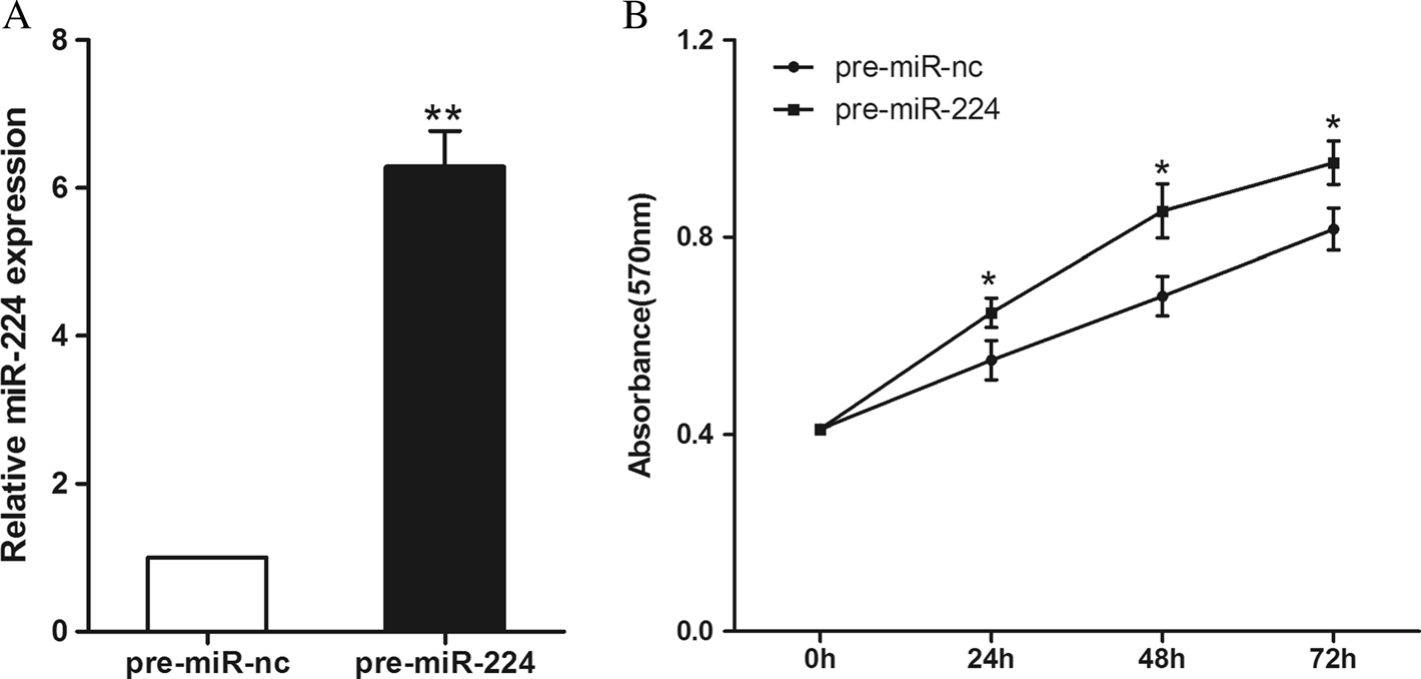
**MiR-224 promotes CRC cell proliferation. (A)** miR-224 expression in SW480 cells transfected with pre-miR-224 or pre-miR-nc. **(B)** MTT assay was performed to determine SW480 proliferation. *P<0.05. **P<0.01.

MiR-224 was upregulated in CRC, implicating its potential role in CRC cells biological properties. To further characterize the functional importance in CRC tumorigenesis, we examined the effect of miR-224 on the proliferation of CRC cells using MTT assay. Compared to pre-miR-nc, transfection with pre-miR-224 in SW480 cells led to an approximately 6-fold increase in miR-224 expression as detected by qRT-PCR (Figure 2A). We observed that overexpression of miR-224 significantly promoted the proliferation of SW480 cells, at 24, 48, 72 h after transfection (*P*<0.05, Figure 2B).

### MiR-224 regulates CRC cell invasion and migration in vitro

**Figure 3:**
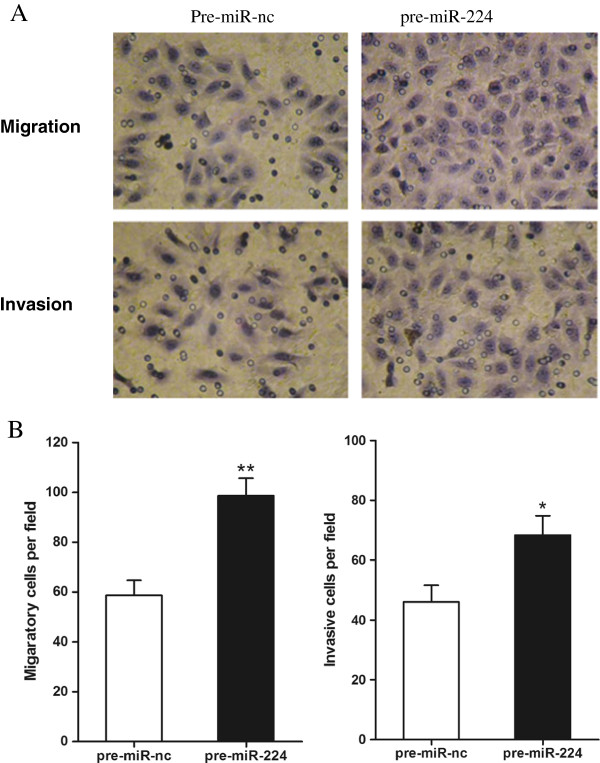
**MiR-224 promotes migration and invation of CRC cells in vitro. (A)** Representative photographs of migratory cells on the membrane of transwell chambers in migration and invasion assays (magnification, ×400). **(B)** The number of migratory/invasive cells was counted per field. *P<0.05, **P<0.01.

The potential roles of miR-224 in CRC cell migration and invasion were assessed using transwell migration and invasion assays. We observed that cell migration was significantly increased following transfection with pre-miR-224 compared with the negative control (*P*<0.01, Figure 3A, 3B). We then examined the effect of miR-224 on cell invasion across an extracellular matrix and showed that in SW480 cells, the overexpression of miR-224 markedly enhanced the invasive potential compared with the control (*P*<0.05, Figure 3A, 3B). These observations suggest that miR-224 plays an important role in promoting migration and invasive ability of CRC cells.

### MiR-224 binds to the 3′UTR of SMAD4

**Figure 4:**
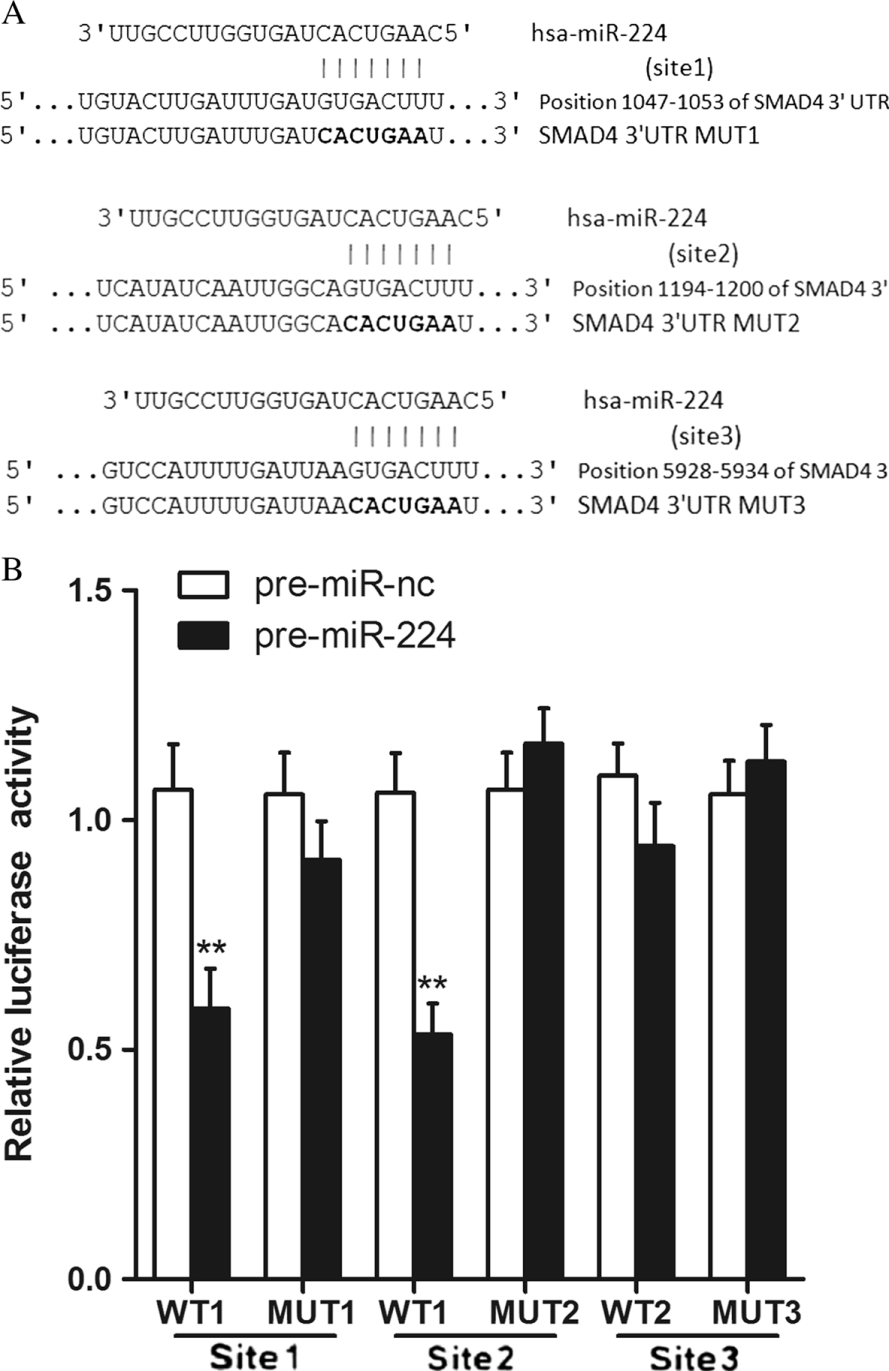
**SMAD4 is a validated target of miR-224. (A)** The wild-type and mutated 3′UTR of SMAD4, with the seed region (site1, site2 and site3) and base substitutions (bold). **(B)** Dual luciferase report assays were performed on HEK 293T cells. Each bar represents mean values ± SD from three independent experiments. **P<0.01.

Analysis by using publicly available programs, TargetScan ( http://www.targetscan.org/) and miRanda ( http://www.microrna.org/microrna/home.do), indicates that SMAD4 is theoretically the target gene of miR-224 (Figure 4A). Therefore, in the current study, we further determined whether SMAD4 gene was an authentic target gene of miR-224 in CRC.

We performed a luciferase reporter assay to verify that miR-224 directly targets SMAD4.Sequences of the 3′UTR of the SMAD4 mRNA surrounding the two close miR-224 potential binding sites (site 1 and site 2) containing the wild-type (WT1).we cloned the regions of 3′UTR each containing one putative miR-224 binding site into the psicheck-2 vector and named as WT1 (site1, site2) and WT2 (site3) (Figure 4A). The reporter constructs harboring mutation of the miR-224 target sites were generated similarly (MUT1, MUT2 and MUT3) (Figure 4A). The luciferase reporter constructs were transfected into HEK 293T cells, along with pre-miR-224 or pre-miR-nc. Luciferase activites were then measured. The luciferase activity of WT1 (site1, site2) reporter transfected with pre-miR-224 was significantly decreased compared with control (*P*<0.01, Figure 4B), while the luciferase activity of the WT2 (site3) reporter was not interfered with after transfection with pre-miR-224 compared with control (*P*>0.05, Figure 4B). These data indicate that miR-224 may target SMAD4 gene through the seeding region of wild type 3′UTR (site1, site2). However, the luciferase reporter activity was not inhibited by miR-224 when the seeding sites were mutated.

### MiR-224 inhibits SMAD4 protein expression but not mRNA level

**Figure 5:**
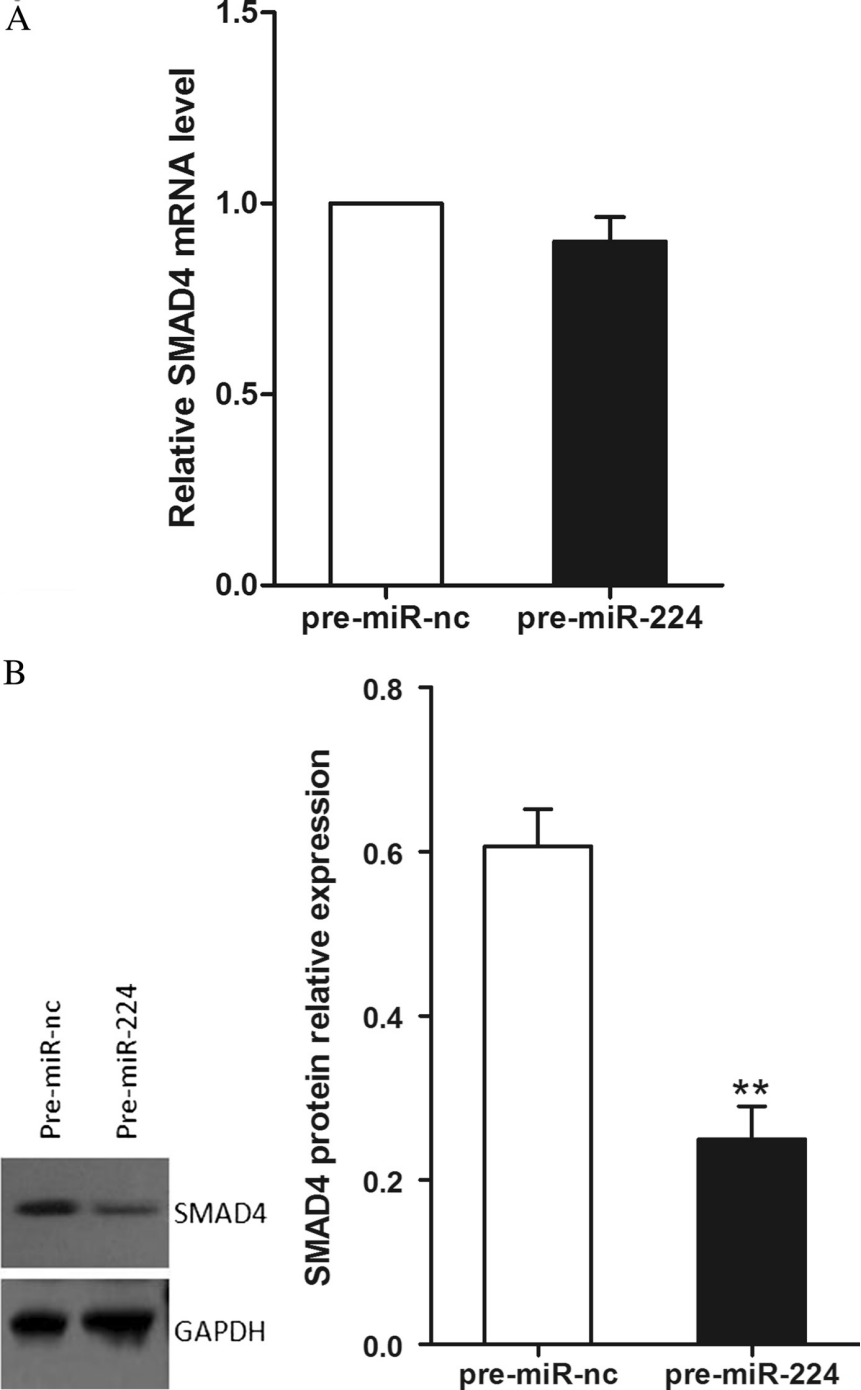
**MiR-224 down-regulates SMAD4 protein expression but not mRNA level. (A)** The expression of SMAD4 mRNA was analyzed by qRT-PCR assay. **(B)** The expression of SMAD4 protein was analyzed by western blot assay. **P<0.01.

To further confirm that SMAD4 was the downstream target of miR-224, we analyzed SMAD4 mRNA and protein levels in transfected SW480 cells by qRT-PCR and Western blot. Western blot analysis demonstrated that high expression of miR-224 dramatically suppressed the endogenous protein level of SMAD4 (*P*<0.01, Figure 5B), while mRNA remained unchanged (*P*>0.05, Figure 5A).Thus, SMAD4 is likely to be suppressed by miR-224 through translational inhibition.

## Disscussion

It was reported that disease relapse was an important factor leading to the poor survival of colorectal cancer patients [17]. At present, poor clinicopathological characteristics and high carcinoembryonic antigen (CEA) level were known as high risk factors for relapse but with varying reliability reported [18]. Therefore, effective biomarkers were wanted to distinguish between patients with and without high relapse risk followed by appropriate therapy in CRC.

Differential miRNA expression in tumor samples compared to normal samples or between groups of tumor samples with a favourable and poor clinical outcome have been used to generate miRNA signatures with potential prognostic and/or predictive value [19,20]. In the current study, we confirmed that miR-224 expression in CRC tumor tissues was significantly higher than that in normal tissues. Furthermore, miR-224 expression levels were significantly up-regulated in the tissues of CRC patients with disease relapse compared with those without disease relapse, and the CRC patients with up-regulated miR-224 in tumor tissues had a high risk of relapse. Thus, miR-224 could function as a potential predictive marker for relapse following radical surgery of colorectal cancer.

In this study, we investigated the biological role of miR-224 in regulating CRC cancer progression. Our results revealed that miR-224 promoted CRC cells growth, migration and invasion in vitro. To address the molecular mechanisms involved in miR-224-mediated changes of biological properties, SMAD4 was selected for further study because it was predicted to be a target of miR-224 by bioinformatics analysis. SMAD4 belongs to the evolutionarily conserved family of SMAD proteins which are transmitters of signals from the transforming growth factor-β (TGF-β) superfamily of cytokines [21]. It is suggested that SMAD4 can function as a tumor suppressor gene in gastrointestinal carcinoma [22,23]. Previous study showed that patients with tumors expressing low SMAD4 levels had significantly worse overall and disease-free survival than patients with high levels in colorectal cancer [24]. Moreover, Loss of SMAD4 expression was found to be associated with liver metastasis, and reduced SMAD4 expression enhances tumorigenicity in CRC [25]. A recent study also reported that loss of SMAD4 promoted migration and invasion, and mediated epithelial– mesenchymal transition (EMT) in CRC cell line SW480 [26,27]. Hence, it is an attractive target for anti-cancer therapy in colorectal cancer.

Our study suggested that SMAD4 was a possible target of miR-224. Firstly, the luciferase reporter assay demonstrated its down-regulation was mediated by the direct binding of miR-224 to the SMAD4 3′-UTR, because the alteration of this region abolished this effect. Secondly, over-expression of miR-224 suppressed SMAD4 protein levels without any change in SMAD4 mRNA expression. Therefore, we proposed that the main mechanism of miR-224-induced SMAD4 suppression was post-transcriptional. In addition, SMAD4 has been confirmed as a target gene of miR-224 in Granulosa Cells [28]. In our study, restoration of miR-224 promoted CRC cell proliferation, migration and invasion, this could possibly be due to miR-224-mediated down-regulation of SMAD4 expression.

Cancer stem cells (CSCs) are predicted to be critical drivers of tumor progression due to CSC characteristics including self-renewal and pluripotency, drug resistance, limitless proliferative potential and metastatic capability, suggesting that targeting CSC characteristics would likely eliminate CSCs which are the “seeds” of tumor recurrence and metastasis. Specific miRNAs have been shown to be involved in CSC regulation in CRC, such as miR-328 and miR-449b [29,30]. Recently, Fellenberg et al. showed that the miR-224 functions as an important regulator of stem cells induction by targeting the apoptosis inhibitor, API5 [31]. The generation of CSCs involves a process of mesenchymal-to-epithelial transition (MET), therefore factors inducing MET or blocking the EMT by inhibiting TGF-β signaling play an essential role in cell reprogramming [32]. It is also known that TGF-β/Smad4 signaling plays a crucial role in the regulation of EMT as well as cell stemness in CRC [27,33]. We have discovered a novel target of miR-224 (Smad4), which has key function in TGF-β signaling, providing the possibility that miR-224 may mediate CSC by suppressing TGF-β/Smad4 activity. Thus, our studies might provide a potential molecular mechanism and crosstalk of CSC regulation and tumor metastasis.

In summary, the association between increased levels of miR-224 and disease relapse in CRC patients indicated that miR-224 was a potential biomarker for identifying high-risk CRC patients after radical resection. The present data showed that miR-224 had oncogenic effects, including the promotion of CRC cell proliferation, migration and invasion, at least in part by targeting the anti-oncogene SMAD4, highlighting the function of miR-224 in the process of tumor progression.

## Competing interests

The authors declare that they have no competing interests.

## Authors’ contributions

ZGJ, XHX and LY performed experiments; ZGJ, ZH and ZT designed research and wrote the paper; ZGJ and ZH analyzed data. All authors read and approved the final manuscript.

## References

[B1] Jemal A Siegel R Ward E Hao Y Xu J Murray T Thun MJ Cancer statistics, 2008 CA Cancer J Clin 2008 58 2 71 96 10.3322/CA.2007.0010 18287387

[B2] Kobayashi H Mochizuki H Sugihara K Morita T Kotake K Teramoto T Kameoka S Saito Y Takahashi K Hase K Characteristics of recurrence and surveillance tools after curative resection for colorectal cancer: a multicenter study Surgery 2007 141 1 67 75 10.1016/j.surg.2006.07.020 17188169

[B3] Bartel DP MicroRNAs: genomics, biogenesis, mechanism, and function Cell 2004 116 2 281 297 10.1016/S0092-8674(04)00045-5 14744438

[B4] Bartel DP MicroRNAs: target recognition and regulatory functions Cell 2009 136 2 215 233 10.1016/j.cell.2009.01.002 19167326 PMC3794896

[B5] Munker R Calin GA MicroRNA profiling in cancer Clin Sci (Lond) 2011 121 4 141 158 10.1042/CS20110005 21526983

[B6] Farazi TA Spitzer JI Morozov P Tuschl T miRNAs in human cancer J Pathol 2011 223 2 102 115 10.1002/path.2806 21125669 PMC3069496

[B7] Lee DY Deng Z Wang CH Yang BB MicroRNA-378 promotes cell survival, tumor growth, and angiogenesis by targeting SuFu and Fus-1 expression Proc Natl Acad Sci USA 2007 104 51 20350 20355 10.1073/pnas.0706901104 18077375 PMC2154434

[B8] Paterson EL Kazenwadel J Bert AG Khew-Goodall Y Ruszkiewicz A Goodall GJ Down-regulation of the miRNA-200 family at the invasive front of colorectal cancers with degraded basement membrane indicates EMT is involved in cancer progression Neoplasia 2013 15 2 180 191 23441132 10.1593/neo.121828 PMC3579320

[B9] Zhang GJ Xiao HX Tian HP Liu ZL Xia SS Zhou T Upregulation of microRNA-155 promotes the migration and invasion of colorectal cancer cells through the regulation of claudin-1 expression Int J Mol Med 2013 31 6 1375 1380 23588589 10.3892/ijmm.2013.1348

[B10] Shen SN Wang LF Jia YF Hao YQ Zhang L Wang H Upregulation of microRNA-224 is associated with aggressive progression and poor prognosis in human cervical cancer Diagn Pathol 2013 8 69 10.1186/1746-1596-8-69 23631806 PMC3661379

[B11] Mees ST Mardin WA Sielker S Willscher E Senninger N Schleicher C Colombo-Benkmann M Haier J Involvement of CD40 targeting miR-224 and miR-486 on the progression of pancreatic ductal adenocarcinomas Ann Surg Oncol 2009 16 8 2339 2350 10.1245/s10434-009-0531-4 19475450

[B12] Huang L Dai T Lin X Zhao X Chen X Wang C Li X Shen H Wang X MicroRNA-224 targets RKIP to control cell invasion and expression of metastasis genes in human breast cancer cells Biochem Biophys Res Commun 2012 425 2 127 133 10.1016/j.bbrc.2012.07.025 22809510

[B13] Zhang Y Takahashi S Tasaka A Yoshima T Ochi H Chayama K Involvement of microRNA-224 in cell proliferation, migration, invasion, and anti-apoptosis in hepatocellular carcinoma J Gastroenterol Hepatol 2013 28 3 565 575 10.1111/j.1440-1746.2012.07271.x 22989374

[B14] Motoyama K Inoue H Takatsuno Y Tanaka F Mimori K Uetake H Sugihara K Mori M Over- and under-expressed microRNAs in human colorectal cancer Int J Oncol 2009 34 4 1069 1075 19287964 10.3892/ijo_00000233

[B15] Arndt GM Dossey L Cullen LM Lai A Druker R Eisbacher M Zhang C Tran N Fan H Retzlaff K Characterization of global microRNA expression reveals oncogenic potential of miR-145 in metastatic colorectal cancer BMC cancer 2009 9 374 10.1186/1471-2407-9-374 19843336 PMC2770572

[B16] Mencia N Selga E Noe V Ciudad CJ Underexpression of miR-224 in methotrexate resistant human colon cancer cells Biochem Pharmacol 2011 82 11 1572 1582 10.1016/j.bcp.2011.08.009 21864507

[B17] Li S Gao J Gu J Yuan J Hua D Shen L MicroRNA-215 inhibits relapse of colorectal cancer patients following radical surgery Medical oncology 2013 30 2 549 23532818 10.1007/s12032-013-0549-0

[B18] Tsai HL Chu KS Huang YH Su YC Wu JY Kuo CH Chen CW Wang JY Predictive factors of early relapse in UICC stage I-III colorectal cancer patients after curative resection J Surg Oncol 2009 100 8 736 743 10.1002/jso.21404 19757443

[B19] Garzon R Marcucci G Potential of microRNAs for cancer diagnostics, prognostication and therapy Curr Opin Oncol 2012 24 6 655 659 10.1097/CCO.0b013e328358522c 23079782

[B20] Albulescu R Neagu M Albulescu L Tanase C Tissular and soluble miRNAs for diagnostic and therapy improvement in digestive tract cancers Expert Rev Mol Diagn 2011 11 1 101 120 10.1586/erm.10.106 21171925

[B21] Massague J Chen YG Controlling TGF-beta signaling Genes Dev 2000 14 6 627 644 10733523

[B22] Wang LH Kim SH Lee JH Choi YL Kim YC Park TS Hong YC Wu CF Shin YK Inactivation of SMAD4 tumor suppressor gene during gastric carcinoma progression Clin Cancer Res 2007 13 1 102 110 10.1158/1078-0432.CCR-06-1467 17200344

[B23] Maitra A Molberg K Albores-Saavedra J Lindberg G Loss of Dpc4 expression in colonic adenocarcinomas correlates with the presence of metastatic disease Am J Pathol 2000 157 4 1105 1111 10.1016/S0002-9440(10)64625-1 11021814 PMC1850169

[B24] Alazzouzi H Alhopuro P Salovaara R Sammalkorpi H Jarvinen H Mecklin JP Hemminki A Schwartz S Jr Aaltonen LA Arango D SMAD4 as a prognostic marker in colorectal cancer Clin Cancer Res 2005 11 7 2606 2611 10.1158/1078-0432.CCR-04-1458 15814640

[B25] Zhang B Halder SK Kashikar ND Cho YJ Datta A Gorden DL Datta PK Antimetastatic role of Smad4 signaling in colorectal cancer Gastroenterology 2010 138 3 969 980 e961-963 10.1053/j.gastro.2009.11.004 19909744 PMC2831103

[B26] Muller N Reinacher-Schick A Baldus S van Hengel J Berx G Baar A van Roy F Schmiegel W Schwarte-Waldhoff I Smad4 induces the tumor suppressor E-cadherin and P-cadherin in colon carcinoma cells Oncogene 2002 21 39 6049 6058 10.1038/sj.onc.1205766 12203117

[B27] Pohl M Radacz Y Pawlik N Schoeneck A Baldus SE Munding J Schmiegel W Schwarte-Waldhoff I Reinacher-Schick A SMAD4 mediates mesenchymal- epithelial reversion in SW480 colon carcinoma cells Anticancer Res 2010 30 7 2603 2613 20682989

[B28] Yao G Yin M Lian J Tian H Liu L Li X Sun F MicroRNA-224 is involved in transforming growth factor-beta-mediated mouse granulosa cell proliferation and granulosa cell function by targeting Smad4 Mol Endocrinol 2010 24 3 540 551 10.1210/me.2009-0432 20118412 PMC5419098

[B29] Xu XT Xu Q Tong JL Zhu MM Nie F Chen X Xiao SD Ran ZH MicroRNA expression profiling identifies miR-328 regulates cancer stem cell-like SP cells in colorectal cancer Br J Cancer 2012 106 7 1320 1330 10.1038/bjc.2012.88 22453125 PMC3314795

[B30] Fang Y Gu X Li Z Xiang J Chen Z miR-449b inhibits the proliferation of SW1116 colon cancer stem cells through downregulation of CCND1 andE2F3 expression Oncol Rep 2013 30 1 399 406 23674142 10.3892/or.2013.2465

[B31] Fellenberg J Saehr H Lehner B Depeweg D A microRNA signature differentiates between giant cell tumor derived neoplastic stromal cells and mesenchymal stem cells Cancer Lett 2012 321 2 162 168 10.1016/j.canlet.2012.01.043 22326282

[B32] Li R Liang J Ni S Zhou T Qing X Li H He W Chen J Li F Zhuang Q A mesenchymal-to-epithelial transition initiates and is required for the nuclear reprogramming of mouse fibroblasts Cell Stem Cell 2010 7 1 51 63 10.1016/j.stem.2010.04.014 20621050

[B33] Roy S Majumdar AP Signaling in colon cancer stem cells J Mol Signal 2012 7 1 11 10.1186/1750-2187-7-11 22866952 PMC3485105

